# Screening of synthetic 1,2,3-triazolic compounds inspired by SRPIN340 as anti-*Trypanosoma cruzi* agents

**DOI:** 10.1590/0037-8682-0585-2023

**Published:** 2024-07-29

**Authors:** Fernanda Karoline Vieira da Silva Torchelsen, Tamiles Caroline Pedrosa Fernandes, Sara Maria Ribeiro de Sousa, Policarpo Ademar Sales-Junior, Renata Tupinambá Branquinho, Silvane Maria Fonseca Murta, Róbson Ricardo Teixeira, Vanessa Carla Furtado Mosqueira, Marta de Lana

**Affiliations:** 1 Universidade Federal de Ouro Preto, Programa de Pós-Graduação em Ciências Farmacêuticas, Ouro Preto, MG, Brasil.; 2 Universidade Federal de Ouro Preto, Programa de Pós-Graduação em Ciências Biológicas, Núcleo de Pesquisas em Ciências Biológicas, Ouro Preto, MG, Brasil.; 3 Universidade Federal de Viçosa, Departamento de Química, Viçosa, MG, Brasil.; 4 Instituto René-Rachou/Fiocruz Minas, Belo Horizonte, MG, Brasil.

**Keywords:** Chagas disease, Triazoles, Screening, Chemotherapy

## Abstract

**Background:**

The current treatments for Chagas disease (CD) include benznidazole and nifurtimox, which have limited efficacy and cause numerous side effects. Triazoles are candidates for new CD treatments due to their ability to eliminate *T. cruzi* parasites by inhibiting ergosterol synthesis, thereby damaging the cell membranes of the parasite.

**Methods:**

Eleven synthetic analogs of the kinase inhibitor SRPIN340 containing a triazole core (compounds **6A**-**6K**) were screened *in vitro* against the Tulahuen strain transfected with β-galactosidase, and their IC50, CC50, and selectivity indexes (SI) were calculated. Compounds with an SI > 50 were further evaluated in mice infected with the *T. cruzi* Y strain by rapid testing.

**Results:**

Eight compounds were active *in vitro* with IC50 values ranging from 0.5-10.5 µg/mL. The most active compounds, **6E** and **6H**, had SI values of 125.2 and 69.6, respectively. These compounds also showed *in vivo* activity, leading to a reduction in parasitemia at doses of 10, 50, and 250 mg/kg/day. At doses of 50 and 250 mg/kg/day, parasitemia was significantly reduced compared to infected untreated animals, with no significant differences between the effects of **6E** and **6H**.

**Conclusions:**

This study identified two new promising compounds for CD chemotherapy and confirmed their activity against *T. cruzi*.

## INTRODUCTION

Chagas disease (CD), caused by the obligate hemoflagellate protozoan parasite *Trypanosoma cruzi*, was first identified over a century ago^1^. Transmission of CD occurs through various routes, including vector-borne transmission via blood-sucking triatomine insects, ingestion of contaminated food, blood transfusion, organ transplantation, and congenital transmission^2^. The disease is endemic to 21 countries, primarily in Latin America^3^, affecting approximately six million people. However, CD has extended its reach to several countries across different continents due to the migration of infected individuals, leading to transmission mechanisms independent of triatomine vectors^4^ ^,^ ^5^. CD typically manifests in an initial acute phase, progressing into an asymptomatic chronic phase for survivors of the acute stage. Approximately 30-35% of chronically infected individuals develop cardiac or digestive alterations, such as megaesophagus and megacolon^6^. Furthermore, disease reactivation in chronically infected patients often occurs in immunodeficient states, such as those induced by transplantation^7^, lupus^8^ ^,^ ^9^, and HIV infections^10^. The reactivation of CD in HIV-infected patients poses particular challenges, with an estimated 16,100 reported cases, comprising approximately 1.3-5% of patients living with *T. cruzi* infection^11^. This subset of patients commonly exhibits more severe clinical manifestations, including central nervous system involvement^10^ ^,^ ^12^.

Since its discovery, there have been persistent efforts to find effective treatments for all clinical stages of CD, particularly late chronic infections, which pose significant challenges. Benznidazole (BZ) and nifurtimox (NF) are currently the only drugs available for CD treatment in humans^13^. While both drugs achieve a significant cure rate in acute cases and recent infections, they are associated with numerous side effects, leading to treatment discontinuation, especially in adults^14^. Additionally, low cure rates have been reported in the later chronic phase^14^, including immunosuppressed patients who may experience reactivation and severe clinical features^15^. Consequently, ongoing research focuses on identifying better treatment options, including drug repositioning^16^, exploration of natural products^17^, and the development of new drugs^18^ targeting specific *T. cruzi* biomolecular targets*.*

In recent years, several clinical trials have investigated modifications to the benznidazole (BZ) regimen, including BENDITA^19^, MULTIBENZ^20^, and BETTY^21^. While numerous drugs targeting *Trypanosoma cruzi* have undergone preclinical testing^22^ ^,^ ^23^, only a limited number have progressed to human trials. Notably, fexinidazole, a re-evaluated molecule licensed for Human American trypanosomiasis, underwent assessment of its risk-benefit profile for treating adult patients in the chronic phase of Chagas disease^19^. Among the various classes of compounds that target *T. cruzi*, 1,2,3-triazoles have emerged as significant^17^ ^-^ ^20^, including itraconazole^24^, posaconazole^25^, and the prodrug ravuconazole (E1224)^26^, which have progressed to clinical trials. The inclusion of 1,2,3-triazoles in clinical trials and their relevance in Chagas disease (CD) treatment studies appear to stem from their primary mechanism of action, which involves inhibiting the 14α-sterol-demethylase enzyme, thereby preventing ergosterol binding^27^ and ultimately leading to the fatal disruption of the parasite membrane.

In Brazil, reports on azole derivatives have shown inconsistency. While one clinical study demonstrated the efficacy of azoles, including itraconazole and ketoconazole, in reducing parasitemia, particularly in cases of CD reactivation in immunosuppressed patients with HIV^28^, other study^29^ reported failure when evaluating itraconazole as an alternative for chronically infected patients. Despite this variability, both approaches share the common goal of substituting benznidazole due to its adverse effects. In contrast, in Chile, the clinical experience with itraconazole and allopurinol in patients with the chronic phase of CD is more promising, with their use leading to parasitological cure in patients treated with itraconazole^24^ ^,^ ^30^. Additionally, there are reports of posaconazole successfully treating Chagas Disease reactivation in a patient with comorbid systemic lupus erythematosus^31^.

Azoles continue to be investigated in studies exploring their combinations with BZ, such as posaconazole in the STOPCHAGAS clinical trials^32^ and fosravuconazole in the BENDITA trial^33^. 

Considering the relevance of azoles in the pursuit of new active drugs against CD, this study evaluated the *in vitro* and *in vivo* anti-*T. cruzi* activity of 11 synthetic 1,2,3-triazole compounds inspired by the kinase inhibitor SRPIN340, which inhibits glioblastoma proliferation *in vitro* ^34^. 

## METHODS

### ● Substances

**FIGURE 1:**
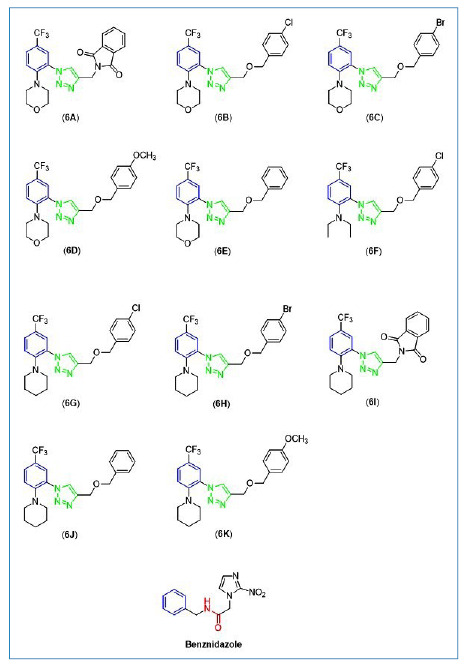
Structures of benznidazole and 1,2,3-triazole compounds evaluated against *Trypanosoma cruzi* in the screening assays.

 The compounds **6A-6K** were evaluated as anti-*T. cruzi* agents, were prepared and structurally characterized as previously described by Sousa et al. Their structures are depicted in Figure 1.

 In comparison to BZ (Figure 1), a compound clinically utilized for treating Chagas' Disease, compounds **6A**-**6K** feature a tri-substituted aromatic ring (highlighted in blue in Figure 1), while BZ presents a monosubstituted one. Additionally, BZ possesses an amide substituent (highlighted in red in Figure 1), whereas compounds **6A**-**6K** feature a 1,2,3-triazole functionality (highlighted in green in Figure 1). Notably, the 1,2,3-triazole group acts as a bioisostere of an amide functionality. Although both BZ and compounds **6A**-**6K** fall under the category of azoles, the former is an imidazole, whereas the latter are 1,2,3-triazoles. 1,2,3-Triazoles constitute a class of five-membered ring heterocyclic compounds widely utilized in medicinal chemistry. They possess the ability to form hydrogen bonds and participate in dipole-dipole and pi-stacking interactions, thus enhancing solubility and binding affinity to molecular targets. Furthermore, this structural framework typically exhibits resistance to acidic and basic hydrolysis under oxidizing and reducing conditions^35^. Due to these advantages, the 1,2,3-triazole structural motif has been extensively employed in designing compounds with various biological activities^35^ ^-^ ^38^, including trypanocidal agents^39^ ^-^ ^42^. Compounds **6A**-**6K** were synthesized to assess the influence of diverse groups attached to the aromatic ring (highlighted in blue in Figure 1) and the 1,2,3-triazole functionality on bioactivity.

###  ● Anti-*T. cruzi* evaluation *in vitro*

 The *in vitro* assays were conducted at the Centro de Pesquisas René Rachou-Fiocruz (FIOCRUZ-MINAS), Minas Gerais, Brazil. To evaluate trypanocidal activity, initial *in vitro* screening was performed following the protocols described by Buckner et al.^43^ and Romanha et al*.* ^44^. In this assay, Tulahuen *T. cruzi* strain expressing β-galactosidase trypomastigotes and intracellular amastigotes were cultured in mouse L929 fibroblasts in RPMI1640 medium without phenol red and containing 10% fetal bovine serum. Tissue culture microplates with L929 fibroblasts were incubated overnight at 37°C with 5% CO_2_. Subsequently, the Tulahuen β-galactosidase-expressing trypomastigotes were added at a ratio of 1:10 cells to trypomastigotes for 2 hours. The medium was then replaced with fresh medium, and the cultures were incubated for 48 hours. Following this step, the medium was replaced with a solution of the test compound, diluted in dimethyl sulfoxide (DMSO, less than 1%), in RPMI1640 medium with concentrations ranging from 5 to 20 µM. After seven days of incubation, a solution containing 100 µM chlorophenol red β-D-galactopyranoside and 0.1% Nonidet P-40 was added and incubated overnight, after which absorbance was assessed at 570 nm. Benznidazole was utilized as a positive control at 1 µg/mL (3.84 µM). 

Because the amount of beta-galactosidase is directly proportional to the number of parasites, trypanocidal activity was assessed by measuring the decrease in beta-galactosidase activity in the treated cultures compared to the infected control culture without treatment. To determine the cytotoxicity of the compounds on L929 cells, absorbance was measured at two wavelengths (570 and 600 nm) after 4-6 hours of incubation with alamarBlue® reagent (Invitrogen Corporation, USA). Cytotoxicity was evaluated by analyzing the difference in reagent reduction between treated cells (TC) and untreated cells (UT) using an equation provided by the manufacturer of alamarBlue®:

$$\begin{array}{rr} & \;(117,216)\text{(Abs570 TC) -}(80,586)\text{(Abs600 TC) X}100\\ & \;(117,216)\text{(Abs570 UT) - (80,586) (Abs600 UT)} \end{array}$$

Half-maximum inhibitory concentration (IC50) and half-maximum cytotoxicity concentration (CC50) values were determined through linear interpolation^45^ using Excel software (Microsoft Corporation, USA). The assay was conducted in triplicate, and the results are expressed as the percentage inhibition of parasite growth Cytotoxicity assay.

*In vitro* cytotoxicity assessments of the compounds were performed using AlamarBlue®. The cells and experimental conditions mirrored those used for the trypanocidal assay. After a 96-hour exposure of mouse L929 fibroblasts infected with *T. cruzi* to the test compounds, alamarBlue® was added, and absorbance was measured after an additional 4-6-hour incubation. IC50 values were determined by linear interpolation, and the selectivity index (SI) was calculated as the ratio of CC50 (the concentration causing 50% cytotoxicity) to IC50 (the concentration causing 50% parasitic inhibition). Benznidazole served as the positive control for comparative analysis.

###  ● Anti-*T. cruzi* evaluation *in vivo*

 The *in vivo* assays were conducted at the Laboratório de Pesquisas Clínicas, Escola de Farmácia, Universidade Federal de Ouro Preto (UFOP), Minas Gerais State, Brazil, utilizing only compounds with a selectivity index (SI) exceeding 50 (Table 1).

**TABLE 1:** Trypanocidal activity, cytotoxicity, and selectivity index (SI) of 1,2,3-triazole compounds 6A-K and benznidazole.

Compound	IC_50_ µg/mL (µM)	CC_50_ µg/mL (µM)	SI
6A	Not active	-	-
6B	2.3 (5.0)	36.1 (79)	15.7
6C	3.4 (6.8)	80.0 (161.3)	23.5
6D	6.6 (15.7)	>20.0 (47.6)	>3.0
6E	0.5 (1.3)	62.6 (160)	125.2
6F	Not active	-	-
6G	4.7 (10.4)	>160 (355)	34.0
6H	2.3 (4.6)	>160 (323)	69.6
6I	Not active	-	-
6J	6.5 (15.6)	>20.0 (48)	6.9
6K	10.5 (23.5)	>20.0 (45)	3.1
Benznidazole	1.0 (3.81)	625 (2381)	625

**IC**_50_ **:** Inhibitory concentration at 50% of intracellular amastigotes of *Trypanosoma cruzi* Tulahuen transfected with β-galactosidase strain growth inhibition; **CC**_50_ **:** cytotoxic concentration at 50% cell viability of mice fibroblast L929. **Selectivity index:** CC_50_/IC_50_. The assayed concentrations were constrained by the compound's solubility in the culture medium, spanning from 20 to 160 µg/mL; **(-):** not determined.

Swiss female mice aged 28-30 days were obtained from the Centro de Ciência Animal of the Universidade Federal de Ouro Preto (UFOP), Minas Gerais State, Brazil. The animals were housed in a temperature range of 20-24 °C with a 12-hour light-dark cycle, and had ad libitum access to filtered water and commercial feed. The experimental procedures were approved by the institutional Comitê de Ética em Experimentação Animal (CEUA-UFOP), Minas Gerais State, Brazil, Protocol 2157041219.

The mice were intraperitoneally infected with 1x10^4^ blood trypomastigotes of the *T. cruzi* Y strain obtained from mice maintained by successive blood passages (CEUA 2015/50). Infection was confirmed by fresh blood examination (FBE)^46^ on the 4^th^ day after infection, and treatment commenced. For the *in vivo* test, the highest concentration attainable when blending the test compounds in various orally safe vehicles was selected. Canola oil yielded the most favorable results. The compounds were diluted with canola oil to the highest possible concentration of 25 mg/mL. Subsequently, two different dilutions (5x) were prepared, resulting in doses of 250, 50, and 10 mg/kg/day. These doses were administered via oral gavage once daily (group n = 8). In parallel, a control group of mice remained infected and untreated (INT), while another group received treatment with the reference drug (BZ) at a dose of 100 mg/kg. The BZ tablets were crushed with 0.5% gum arabic, and suspended in distilled water^47^.

To evaluate the effectiveness of the compounds in reducing parasitemia, FBE was conducted daily for five consecutive days, during which parasitemia was counted^46^. 

## RESULTS

###  ● *In vitro* biological assays 

**FIGURE 2:**
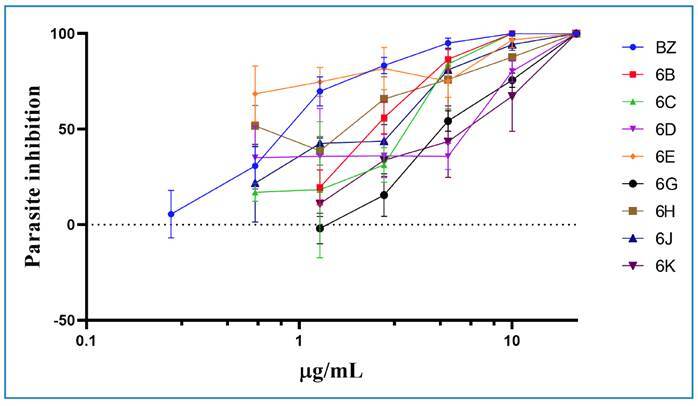
*In vitro* trypanocidal activity evaluation of active 1,2,3-triazole compounds against amastigotes of *Trypanosoma cruzi* Tulahuen transfected with β-galactosidase strain after 96 hours of exposition.

The findings illustrated in Figure 2 highlight that 8 out of the 11 tested compounds demonstrated significant activity against the proliferative stages of *T. cruzi*. The IC50 values of these compounds ranged from 0.5 to 10.47 µg/mL.

 Compound **6E** exhibited the lowest calculated IC_50_ value (0.50 μg/mL or 1.3 µM), indicating it was twice as potent as BZ in terms of mass concentration, which exhibited an IC_50_ of 1 µg/mL (3.81 µM). Additionally, compounds **6B**, **6C**, **6G**, and **6H** showed IC_50_ values below 5 µg/mL (as indicated in **Table 1**). To ensure safety, cytotoxicity assessments were conducted for all active compounds. Compounds **6G** and **6H** exhibited the highest CC_50_ values, surpassing 160 µg/mL (355 and 323 µM, respectively), with the exact value unknown due to the limited solubility of the compounds in the cell culture media. Importantly, none of the compounds demonstrated greater cytotoxicity than BZ, which had a CC_50_ of 625 µg/mL (2.381 µM). 

 The selectivity index (SI), calculated as CC50/IC50, was determined for all active compounds. Compounds with SI values exceeding 50 were identified as promising candidates for *in vivo* experiments, following the guidelines outlined by Romanha et al.^44^ Consequently, compounds **6E** and **6H**, with SI values of 125.2 and 69.6, respectively, were selected for further assessment.

###  ● *In vivo* assay 

**FIGURE 3:**
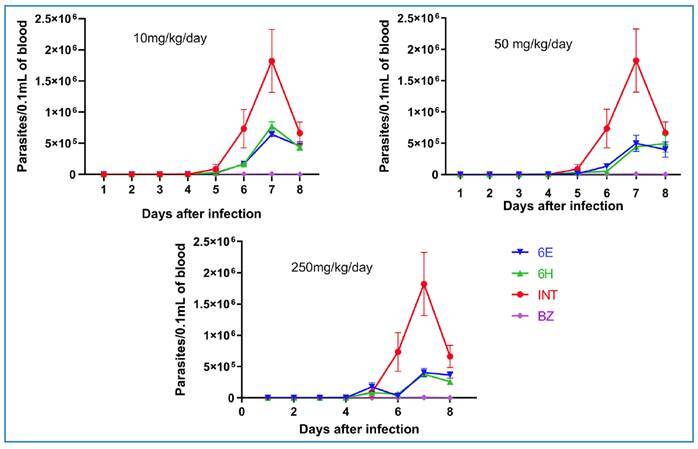
Parasitemia curves of *Swiss* mice infection with *Trypanosoma cruzi* Y strain treated orally with the compounds **6E**, **6H,** and benznidazole for five consecutive days at acute phase of infection.

 Examination of the parasitemia curve depicted in Figure 3 revealed that compounds **6E** and **6H** effectively reduced the parasite count in the bloodstream of mice at doses of 10, 50, and 250 mg/kg/day. 

Notably, the reduction was most pronounced at higher doses compared to untreated animals. Importantly, no discernible differences were observed in the effectiveness of the two compounds.

DISCUSSION

Since the discovery of Chagas disease (CD) by Chagas in 1909, finding an effective cure for all stages of the disease, particularly the late chronic phase, has posed a persistent challenge. Benznidazole has been the primary treatment option since its introduction in the 1970s. Various factors contribute to the difficulty of eradicating the parasite from the host, including the natural resistance of *T. cruzi* strains to existing drugs^48^, the balance of host immunity, nutritional status, comorbidities, the profile of the active drug^49^, and the treatment regimen^14^ ^,^ ^50^. 

Benznidazole treatment is effective during the acute phase of CD. However, diagnosis during this phase is often challenging due to underdiagnosis, as clinical suspicion is not always confirmed by laboratory tests^51^ ^,^ ^52^. In the chronic phase, while benznidazole can improve a patient's quality of life, complete remission of infection is rarely achieved. Moreover, treatment regimens are frequently interrupted due to side effects, which can include dermatitis, fever, lymphadenopathy, bone marrow depression, thrombocytopenic purpura, and polyneuropathy^14^. These adverse effects are particularly relevant in cases of disease reactivation, where patients typically present with compromised health states due to immunocompromised conditions and comorbidities^9^ ^,^ ^10^ ^,^ ^53^. 

The demand for novel drug options for CD arises from the need to address the diverse physiological and health profiles of patients, as well as the various phases and manifestations of the disease. It is crucial to explore safer treatment alternatives, whether aimed at complete eradication of the infection or management and control, to enhance the overall health status of patients.

Therefore, addressing CD requires multiple approaches and strategies. One promising strategy involves exploring specific metabolic interactions between the parasite and host, leading to the evaluation of various compounds under experimental conditions. Among these, inhibitors of the ergosterol pathway have shown promise, including commercially available antifungal drugs such as ketoconazole, fluconazole, and itraconazole^54^. More recently, triazoles, such as ravuconazole (Bristol-Myers Squibb), known for their potent antifungal properties, have demonstrated comparable or superior results to other options such as posaconazole^55^.

In Brazil, clinical experience with immunocompromised patients has demonstrated the successful use of itraconazole and ketoconazole^28^ effectively reducing parasitemia when benznidazole is not tolerated by patients. Conversely, in Chile, treatment with itraconazole and allopurinol^24^ ^,^ ^56^ has led to significant reductions in parasitological test positivity and improvements or prevention of electrocardiographic abnormalities in humans, as reported by Apt et al*.* ^24^ ^,^ ^30^. In contrast, Brener et al*.* ^57^, Lauria-Pires et al*.* ^58^ and Moreira et al.^29^ have shown that ketoconazole, ketoconazole^57^, allopurinol^52^, and itraconazole^29^ are ineffective in eradicating infection in humans. This controversy may stem from differences in individual patient characteristics, including the presence of comorbidities such as HIV^11^ ^,^ ^28^ interplay between drug efficacy and the immune system^59^. Variations in drug dosage and disease phase also play a role. For instance, itraconazole administered at a dose of 400 mg/day for 90 days resulted in parasitemia reduction in CD reactivation cases in patients with HIV, as demonstrated by Almeida et al^28^. However, Moreira et al.^29^ reported treatment failure at lower doses (100 and 200 mg/day for 90 days) in chronic patients. Furthermore, disparities in experience between Brazil and Chile may be attributed to variations in the distribution of *T. cruzi* discrete typing units (DTU)^60^. This variation can influence treatment resistance or susceptibility, as different DTUs may respond differently to the same treatment regimens^61^.

 In our study, the majority of the tested 1,2,3-triazoles (8 out of 11) demonstrated activity against *T. cruzi in vitro*. Two compounds, **6E** (IC_50_=0.5 µg/mL or 1.3 µM) and **6H** (IC_50_= 2.3 µg/mL or 4.6 µM), stood out for their anti-*T. cruzi* activity and selectivity were further evaluated *in vivo*.

 Examination of the structures of the compounds investigated (Figure 1) and the results shown in Table 1 revealed that the presence of a 2-methylisoindoline-1,3-dione fragment attached to the 1,2,3-triazole ring (as seen in compounds **6A** and **6I**) and aliphatic groups connected to nitrogen (**6F**) did not contribute positively to their trypanocidal activity, as these compounds were found to be inactive. In contrast, the active compounds (**6B-6E**) share a common structural feature characterized by the presence of morpholine and benzyloxymethyl fragments. Furthermore, the addition of an electron-donating methoxy group at the benzyloxy ring para position resulted in reduced anti-*T. cruzi* activity compared with compounds featuring electron-withdrawing chlorine and bromine groups. In compounds **6B** and **6C**, the presence of a more electronegative chlorine atom, rather than bromine, confers greater potency against *T. cruzi*. Notably, **6E**, one of the most active compounds, contained no substituents in the benzyloxy group.

 Active substances containing piperidine fragments (**6G**, **6H**, **6J**, and **6K**) exhibited a different trend compared to compounds with morpholine fragments. For instance, compound **6H**, with a *para*-bromine atom attached to its benzyloxy functionality, showed enhanced trypanocidal activity compared to its chlorine counterpart. Interestingly, **6G**, featuring an unsubstituted benzyloxy group, was not the most active compound among those with piperidine fragments.

 A notable observation was that the presence of a para-methoxy group in the benzyloxy moiety resulted in the compounds with the lowest activity in each series.

 In terms of cytotoxicity, our investigation revealed that compounds with a morpholine group exhibited higher toxicity compared to those with a piperidine group. The impact on cell viability varied when these compounds were applied to different cellular lineages and disease models. Notably, the cytotoxicity observed for compound **6A** on L929 cells differed from its effect on the U87MG lineage, where there was no perceived cytotoxicity and cellular viability even showed an increase^34^. Extending our analysis to other compounds examined by Sousa et al.^34^, namely **6B** to **6K** (identified by Sousa et al.^34^ as compounds 8 to 17), we found that these compounds did not substantially affect cellular growth of the U87MG lineage. This discrepancy in cytotoxicity can be primarily attributed to the choice of cell lineage, with U87MG cells representing human glioblastoma cells and L929 cells representing mouse fibroblasts. Given that L929 is a standard cell line for *T. cruzi* screening activity^62^, we opted to use the same uninfected cell line to assess cell viability.

 Surprisingly, the *in vivo* study did not reflect the *in vitro* differences in the trypanocidal activities of the compounds, illustrating the challenge of translational studies^63^. The molecular distinctions between the two studied compounds (**6E**, SI = 101; **6H**, SI = 69) did not significantly affect parasitemia in the animals. Except for the smallest dose tested *in vivo* (10 mg/kg/day) of **6E** and **6H**, which did not markedly reduce parasitemia compared to the untreated group, both compounds appeared to exert their activities independently of the dose, as increasing the dose from 50 to 250 mg/kg/day did not result in improved infection control. Compared with BZ, both compounds exhibited inferior overall performance *in vivo*. However, combination therapy with an active agent that targets the trypomastigote form may lead to improved outcomes in mouse models. These compounds, evaluated *in vivo*, did not demonstrate robust trypanocidal activity during the acute phase of CD and have the potential to augment chronic treatments. Notably, screening outcomes indicated that compounds **6E** and **6H** exhibited substantial activity against the amastigote form, which is prevalent during the chronic stage of the disease.

Moreover, these compounds have potential applications beyond CD. Triazoles, in general, exhibit diverse biological activities, including antibacterial and antifungal properties, as well as, as explored by Sousa et al.^34^, potential anticancer effects. This versatility underscores the broad applicability and potential therapeutic implications of the studied compounds.

In summary, this study highlighted the continued relevance of triazoles in *T. cruzi* drug discovery and identified two promising new compounds for CD chemotherapy research. These potential drug candidates warrant further investigation to assess their effect on the control or cure of *T. cruzi* infection in long-term efficacy studies, in which the compounds, infections, and outcomes would be better monitored.
